# Differences in the Direction of Change of Cerebral Function Parameters Are Evident over Three Years in HIV-Infected Individuals Electively Commencing Initial cART

**DOI:** 10.1371/journal.pone.0118608

**Published:** 2015-02-27

**Authors:** Alan Winston, Rebekah Puls, Stephen J. Kerr, Chris Duncombe, Patrick Li, John M. Gill, Reshmie Ramautarsing, Simon D. Taylor-Robinson, Sean Emery, David A. Cooper, for the ALTAIR Study Group

**Affiliations:** 1 Department of Medicine, Imperial College London, London, United Kingdom; 2 The Kirby Institute, University of New South Wales, Sydney NSW 2052, Australia; 3 HIV NAT, Bangkok, Thailand; 4 Queen Elizabeth Hospital, Hong Kong, Hong Kong; 5 Calgary Health, Alberta, Canada; Rutgers University, UNITED STATES

## Abstract

**Background:**

Changes in cerebral metabolite ratios (CMR) measured on ^1^H-MRS and changes in cognitive function (CF) are described in subjects commencing combination antiretroviral therapy (cART), although the dynamics of such changes are poorly understood.

**Methods:**

Neuroasymptomatic, HIV-infected subjects electively commencing cART were eligible. CMR were assessed in three anatomical voxels and CF assessed at baseline, week 48 and week 144. Overall differences in absolute change in CMRs and CF parameters between 0–48 and 48–144 weeks were assessed.

**Results:**

Twenty-two subjects completed study procedures. Plasma HIV-RNA was <50 copies/mL in all at week 48 and in all, but two subjects at week 144. In general, between weeks 0–48 a rise in N-acetyl-aspartate(NAA)/Creatine(Cr) ratio and a decline in myo-Inositol(mI)/Cr ratio were observed. Between weeks 48–144, small rises in NAA/Cr ratio were observed in two anatomical voxels, whereas a rise in mI/Cr ratio was observed in all anatomical locations (0.31 (0.66) and -0.27 (1.35) between weeks 0–48 and 0.13 (0.91) and 1.13 (1.71) between weeks 48–144 for absolute changes in NAA/Cr and mI/Cr (SD) in frontal-grey voxel, respectively). Global CF score improved between weeks 0–48 and then declined between weeks 48–144 (0.63 (1.16) and -0.63 (0.1.41) for mean absolute change (SD) between weeks 0–48 and weeks 48–144, respectively).

**Conclusions:**

The direction of change of cerebral function parameters differs over time in HIV-infected subjects commencing cART, highlighting the need for long-term follow-up in such studies. The changes we have observed between weeks 48–144 may represent the initial development of cerebral toxicities from cART.

## Introduction

Cerebral function parameters are reported to improve after the initiation of combination antiretroviral therapy (cART). These improvements include changes in measures of cognitive ability [1,2,3,4] and changes in biomarkers of cerebral function, such cerebral metabolites measured using proton magnetic resonance spectroscopy (^1^H-MRS) [5,6,7,8]. However, the temporal trends in changes of cerebral function parameters are poorly understood with most data reporting on observations over short periods of time up to 24 or 48 weeks.

Although such initial improvements in cerebral function parameters are described, on-going cognitive function impairment remains widely reported in effectively treated HIV-infected individuals despite cART [9]. The pathogenesis of this on-going cognitive impairment remains elusive with proposed mechanisms including a lack of suppression of viral replication in sanctuary sites, persisting neuro-inflammatory processes despite cART and neuronal toxicities secondary to antiretroviral agents [10]. Longitudinal clinical data may be of assistance in elucidating the natural history of cerebral function in treated HIV-infected subjects and may assist as supporting data aiding the description of the likely underlying pathogenic mechanisms.

We assessed the dynamics of change in cerebral function parameters in HIV-infected individuals over 144 weeks after electively commencing cART.

## Methods

### Subject selection and study procedures

Neuro-asymptomatic, antiretroviral naïve, HIV-infected subjects attending 4 clinical sites (St. Mary’s Hospital, London, UK; Queen Elizabeth Hospital, Kowloon, Hong Kong; HIVNAT, Bangkok, Thailand; Southern Alberta HIV clinic, Calgary, Canada) entering the ALTAIR study (A Randomised, Open-Label Study Comparing the Safety and Efficacy of Three Different Combination Antiretroviral Regimens as Initial Therapy for HIV Infection, http://clinicaltrials.gov/show/NCT00335322 [11]) were eligible to enter a 3 year sub-study. Ethics approval was granted for all clinical sites in this study and all study participants provided written informed consent.

At study entry, subjects were randomly allocated to commence cART, comprising tenofovir/emtricitabine 300/200 mg once daily with either efavirenz 600 mg once daily (arm1), atazanavir/ritonavir 300/100 mg once daily (arm2), or zidovudine/abacavir 250 or 300 mg twice daily/600 mg once daily (arm3). After 48 weeks, the primary endpoint of the main study revealed poorer virological response rates in arm3 [11], and thereafter all subjects in this arm modified antiretroviral therapy based on investigator choice.

Full study entry criteria have previously been reported [12]. Of note, specific exclusion criteria included current or recent use of antidepressant or antipsychotic therapies, established dementia and viral hepatitis C infection (hepatitis C antibody positive).

### Cerebral ^1^H Magnetic Resonance Spectroscopy

^1^H-MRS was performed on a 1.5 Tesla Phillips Achieva scanner (Imperial College, London, London, UK), a 3.0 Tesla Siemens Avanto scanner (Queen Elizabeth Hospital Site, Kowloon, Hong Kong), a 1.5 Tesla Signa General Electric scanner (HIVNAT site, Bangkok, Thailand) and a 3.0 Tesla Signa Excite scanner (South Alberta HIV clinic, Calgary, Canada) in three anatomical locations as follows; right frontal white matter (FWM), mid-frontal grey matter (FGM) and the right basal ganglia (RBG). Detailed descriptions of the precise anatomical locations are previously reported [12,13]. All spectra were analysed and quantified by one observer (AW) using a Java-based version of the Magnetic Resonance User Interface package (jMRUI Version Number: 3.0) incorporating the AMARES algorithm [14]. Metabolites assessed were N-acetyl-aspartate (NAA), creatine (Cr), choline (Cho) and myo-Inositol (mI). To adjust for different MRI scanners across sites, all metabolites were expressed as cerebral metabolite ratios (CMR) with respect to cerebral Cr.

### Cognitive testing

Cognitive testing was assessed at baseline and weeks 24, 48 and 144. A computerised cognitive test battery was utilised (CogState; Melbourne, Australia). This battery has been described in detail [15] and validated in HIV-disease [16]. This battery contains tasks which are adaptations of standard neuropsychological tests and assess a range of cognitive functions including the following domains: detection, identification, monitoring and matched learning (all assessed via speed of test); associate learning and working memory (assessed via accuracy of test); and executive function (assessed via number of errors made on testing). All study participants completed one full practice test prior to undertaking the study examination in order to minimise learning effects [17].

### Statistical analysis

Statistical analyses were conducted with Stata version 12 (Statacorp, College Station, TX, USA) and analysis conducted according to Cogstate recommendations. Changes in cognitive scores were calculated for each subject, and these scores standardised according to the within-subjects standard deviation (SD). Composite scores were calculated overall and for the speed, accuracy and executive function domains based on the average of standardised scores and composite changes from baseline to week 48 and weeks 48 to 144 were calculated on the average of standardised scores. The global composite score reflects declines in speed and errors, and improvements in accuracy.

Overall differences in absolute change CMRs between 0–48 and 48–144 weeks were assessed. Regression models were used to estimate the mean absolute differences in CMR and composite cognitive scores between 0–48 and 48–144 weeks, and to test whether efavirenz use influenced the changes. In these analyses, P values were adjusted for nine comparisons (3 metabolite ratios in 3 brain regions). Spearman’s rank correlation coefficient was used to test associations between changes in cognitive test scores.

Due to small numbers, all analyses were conducted for the entire patient cohort with no analyses between study treatment arms undertaken. As efavirenz use has been associated with poorer cognitive function [18], the effect of changing to efavirenz after 48 weeks, on cognitive function and CMR, was examined in sensitivity analyses using a random effects regression model. Another sensitivity analysis was undertaken, excluding subjects with a missing plasma viral load at week 144.

## Results

### Patient characteristics

Of 30 subjects enrolled, 22 subjects completed the week 144 study visit. Of those participants who did not continue to the week 144 visit, reasons for study discontinuation were as follows; ^1^H MRS could not be scheduled within the window (n = 2), ^1^H MRS scanner was unavailable (n = 3), ^1^H MRS scan unsuccessful (n = 1) and patients were not contactable (n = 2).

Antiretroviral treatment history and HIV-disease parameters are shown on *Table 1*. Other baseline characteristics have been described in detail previously [12]. Changes in cerebral metabolite ratios and cognitive function for the 30 recruited subjects over 48 weeks have been previously reported [12]. Here we report these changes for the 22 subjects completing 144 weeks of follow up.

**Table 1:** Subject characteristics (n = 22).

Parameter		Value	
*CD4 count*			
Baseline CD4 count, (cells/μL, median / IQR)		238	176–260
Week 48 CD4 count, (cells/μL, median / IQR)		445	274–500
Week 144 CD4 count, (cells/μL, median / IQR)		454	379–664
*HIV-1 RNA*			
HIV-1 RNA baseline, (log_10_ copies/mL, SD)		4.72	0.70
HIV-1 RNA < 50 copies/mL at week 48 (n)		22	
HIV-1 RNA < 50 copies/mL at week 144 (n)		20*	
*Antiretroviral therapy*			
Treatment arm at baseline (number)			
	Arm 1	6	
	Arm 2	8	
	Arm 3	8	
Treatment change at week 48 for Arm 3: Truvada plus (number):			
	atazanavir/r	2	
	darunavir/r	2	
	efavirenz	4	

IQR = interquartile range, SD = standard deviation, ‘r’ = ritonavir

*Data from two patients were missing at week 144

### Changes in cerebral metabolite ratios over 144 weeks

Between weeks 0 and 48, a rise in neuronal CMR (NAA/Cr) and a decline in inflammatory CMR (Cho/Cr and mI/Cr) were observed (*Table 2*). This pattern was observed for all CMR apart from Cho/Cr in the FGM where only a small absolute change over 0–48 weeks was observed.

**Table 2:** Changes in cerebral function parameters over 3 years.

	Details	Mean absolute change				Mean absolute change		
		number	week 0–48	SD	*P-value*	week 48–144	SD	*P-value*
**Cerebral Metabolite Ratio**								
Anatomical area	Ratio							
Frontal Grey	*NAA/Cr*	22	0.31	0.66	0.36	0.13	0.91	1.00
	*Cho/Cr*	22	0.02	0.19	1.00	0.09	0.28	1.00
	*mI/Cr*	21	-0.27	1.35	1.00	1.13	1.71	0.06
Frontal White	*NAA/Cr*	22	0.04	0.74	1.00	0.14	0.77	1.00
	*Cho/Cr*	22	-0.08	0.30	1.00	0.14	0.24	0.09
	*mI/Cr*	21	-0.50	1.54	1.00	1.49	1.49	**0.002**
Right Basal Ganglia	*NAA/Cr*	20	0.64	1.20	0.27	-0.61	1.13	0.27
	*Cho/Cr*	20	-0.09	0.76	1.00	-0.17	0.33	0.27
	*mI/Cr*	20	-0.03	1.05	1.00	0.71	1.46	0.36
**Cognitive test parameter**								
Composite speed score	*decline in score represents improvement*	21	-0.186	0.486	0.10	-0.027	0.452	0.79
Composite accuracy score	*increase in score represents improvement*	21	0.220	0.497	0.06	-0.305	0.499	**0.01**
Executive function score	*decline in score represents improvement*	21	-0.222	0.858	0.25	0.351	1.20	0.19
Global composite score	*increase in score represents improvement*	21	0.627	1.16	**0.02**	-0.629	1.41	0.06

*SD = standard deviation, NAA/Cr = N-acetyl-aspartate/Creatine, Cho/Cr = Choline/Cr, mI/Cr = myo-Inositol/Cr. P*-values <0.05 shown in bold.

**Fig 1:**
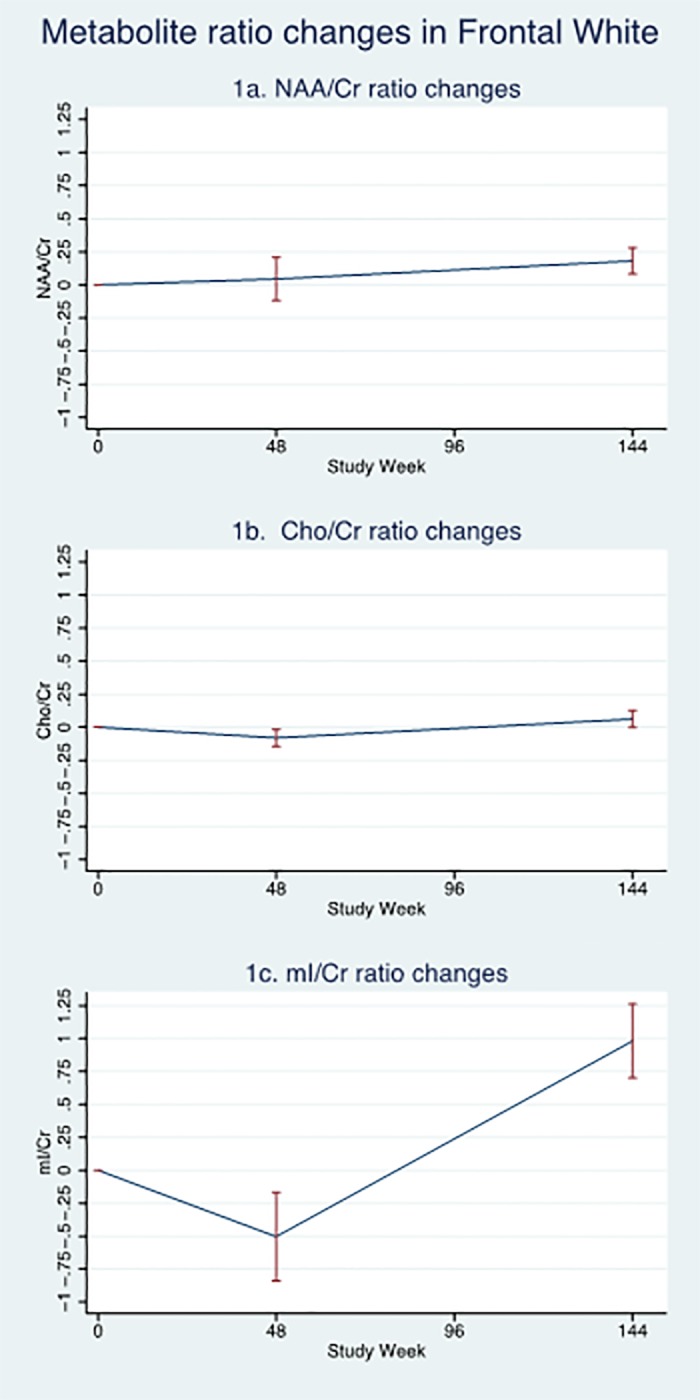
Dynamics of changes in cerebral metabolite ratios in the Frontal White matter over 3 years. Fig. 1A: Mean changes in N-acetyl-aspartate/Creatine ratio; Fig. 1B: Mean changes in Choline/Creatine ratio and Fig. 1C: Mean changes in myo-Inositol/Creatine ratio (error bars represent 1 standard error).

Between weeks 48–144, small increases in neuronal CMR were also observed in the frontal voxels. Conversely, a decline in neuronal CMR was observed in the basal ganglia and a rise in inflammatory CMR observed in all anatomical locations. However, the exception was the Cho/Cr in FGM where only a small absolute change was observed. The dynamics of CMR changes in frontal white matter over time are shown in Fig. 1.

### Changes in cognitive function parameters over 144 weeks

Initially (between weeks 0 to 48), overall cognitive function score improved (mean absolute change in global score of 0.627, *p* = 0.02) and improvement in all composite domains were observed.

Again, on the converse, between weeks 48 to 144 a different pattern was observed. Overall global cognitive score deteriorated (mean absolute change in global score of -0.629, *p* = 0.06) with composite accuracy (-0.305, *p* = 0.01) and executive function (0.351, *p* = 0.19) scores also deteriorating. However there a slight improvement in composite speed observed (-0.027, *p* = 0.79).

### Associations between changes in cerebral function parameters between weeks 48 and 144

Reductions in NAA/Cr ratio (week 48–144) in RBG were associated with an increase in composite speed score change (week 48–144), Spearman’s Correlation r = 0.50, *p* = 0.05. No other statistically significant changes in CMRs between week 48–144 were associated with significant changes in cognitive function parameters (*p*>0.10 all observations).

Of the two sensitivity analyses conducted, the first employed a random effects regression model to investigate whether changes in test parameters could be attributed to changing to an efavirenz containing regimen after week 48. In these analyses, no effect on cognitive test scores was noted in subjects who changed to efavirenz. The Cho/Cr ratio in the FFM was lower (coefficient -0.26 (95%CI -0.48 to -0.05) and mI/Cr in RBG was higher (coefficient 1.24 (0.28 to 2.18) in subjects with a late switch to efavirenz, but these effects were no longer significant after adjusting for multiple comparisons. In the second sensitivity analysis excluding 2 subjects with a missing plasma HIV RNA at week 144, the change coefficients from week 48 to 144 were approximately the same in direction and magnitude for each neurocognitive parameter and metabolite ratio change.

## Discussion

In HIV-infected subjects electively commencing cART for the first time, changes in cerebral function parameters are present and are dynamic with the direction of change differing over time.

In our study we assessed cerebral function parameters which have been widely utilised in this field. The computerised cognitive assessment battery (CogState) has been specifically validated in HIV-disease [16]. Close correlations between measures of cognitive function and measurements of CMR via ^1^H-MRS have been described in HIV-disease historically, prior to the advent of antiretroviral therapy [19], and in more recent years in several stages of the disease course including primary HIV-infection [20], and chronic HIV-infection both prior to the initiation of cART and in subjects on stable therapy [21,22].

We would consider the initial changes in cerebral function parameters observed over 48 weeks to be representative of improvements. For instance, test scores of cognitive function all improved. The changes we observed in cerebral metabolites in the current study may represent a reversal of differences in these parameters which have been reported in untreated HIV-infected subjects compared to matched control populations [5]. However, in common with previous published studies, we do not have imaging data in these individuals prior to HIV-infection to confirm that these changes are a reversal of HIV-associated impairment.

Unlike the improvements observed over the first 48 weeks, there was a decline in measures of cerebral function of the 48 to 144 week period. For instance, global composite cognitive score deteriorated (-0.629, *p* = 0.06). Although this change in composite cognitive score was not statistically significant, this change was greater than 0.5, a *z*-score difference which previous work has suggested would be of clinical relevance [23]. A statistically significant deterioration in cognitive domains measured by accuracy scores was also observed (change 0.499, *p* = 0.01) between week 48 and 144.

To explain this deterioration in cognitive function during weeks 48–144, one could postulate that early on in our study, cognitive testing occurred frequently with a practice test before study entry and then testing at baseline, week 24 and 48. Two years following, only one test was undertaken at week 144 and therefore any practice effect may be less marked. However, there are several counterintuitive arguments to this suggestion. First, subjects did undergo a short practice test prior to undertaking each cognitive assessment on the computerised battery at every visit, including the week 144 visit. Second, a lack of practice effect at the week 144 visit would not explain the changes in CMR observed between week 48–144 and the association between the reduction in NAA/Cr ratio observed in the BRG with change in composite speed score. Therefore, the frequency of test completion is unlikely to explain the observed neurocognitive decline post-week 48.

A further explanation could be related to the type of antiretroviral regimens patients were receiving in our study. Due to arm3 being discontinued after 48 weeks, more subjects were receiving efavirenz by week 144 (n = 10), compared to the number receiving efavirenz (n = 6) at week 48. Use of efavirenz has been associated with poorer measures of cognitive function within a cohort setting [18]. Although a sensitivity analysis does not show that efavirenz use was driving any of our findings, we cannot exclude this cofounder in this small study.

Several causes of variability in cerebral ^1^H MRS measurements, such as inaccurate voxel re-localisation and inter-scanner variability, could have influenced our findings [24]. In order to minimise the effects of voxel re-localisation, during the baseline study scan, voxel placement was based on a study operations manual. Thereafter, on subsequent study scans, voxel placement was undertaken utilising anatomical images of the exact voxel position for each individual subject obtained during the baseline scan. We have minimised any inter-scanner effects by expressing all ^1^H MRS measurable parameters as CMR and conducted sensitivity analyses to ensure no specific scanner in this multicentre study was driving any of the study findings.

Laboratory models using sensitive indices of neural damage have reported toxicities to ensure with many antiretroviral agents in current clinical use [10]. It is possible that the effects we have observed are secondary to such mechanisms. Indeed, the observations we have made on cerebral ^1^H MRS, namely a reduction in neuronal metabolite markers and a rise in inflammatory metabolite markers, would be supportive of this hypothesis. A previous cerebral ^1^H MRS study has reported a reduction in markers of neuronal integrity to be associated with nucleoside-reverse-transcriptase (NRTI) containing antiretroviral therapies in HIV-infected subjects and suggest such changes may be related to mitochondrial toxicities associated with NRTI use [25]. In our study, all subjects received dual-NRTI based antiretroviral regimens, as per standard clinical practice, and of interest 8 subjects allocated to arm3 of the study received triple-NRTI therapy for the first 48 weeks. Our study did not involve measures of antiretroviral drug exposure, either in the plasma or cerebrospinal fluid compartments and therefore we are not able to correlate markers of drug exposure with the cerebral metabolite markers we have measured which in future studies may add further credence to this hypothesis.

Our work will assist in the design of future studies in this field and of importance highlight the need for longitudinal studies to be of sufficient duration to observe not only the initial effects of commencing antiretroviral therapy on cerebral function but to also allow adequate follow-up periods in order to observe the on-going effects cART may have longer term in chronic HIV-disease.
